# Real‐Time Deep‐Learning Image Reconstruction and Instrument Tracking in MR‐Guided Biopsies

**DOI:** 10.1002/jmri.70138

**Published:** 2025-10-01

**Authors:** Constant R. Noordman, Lauren P. W. te Molder, Marnix C. Maas, Christiaan G. Overduin, Jurgen J. Fütterer, Henkjan J. Huisman

**Affiliations:** ^1^ Diagnostic Image Analysis Group, Department of Medical Imaging Radboud University Medical Center Nijmegen the Netherlands; ^2^ Minimally Invasive Image‐Guided Intervention Center, Department of Medical Imaging Radboud University Medical Center Nijmegen the Netherlands; ^3^ Department of Medical Imaging Radboud University Medical Center Nijmegen the Netherlands; ^4^ TechMed Centre University of Twente Enschede the Netherlands

**Keywords:** artificial intelligence, deep learning, image processing (computer‐assisted), interventional radiology, magnetic resonance imaging

## Abstract

**Background:**

Transrectal in‐bore MR‐guided biopsy (MRGB) is accurate but time‐consuming, limiting clinical throughput. Faster imaging could improve workflow and enable real‐time instrument tracking. Existing acceleration methods often use simulated data and lack validation in clinical settings.

**Purpose:**

To accelerate MRGB by using deep learning for undersampled image reconstruction and instrument tracking, trained on multi‐slice MR DICOM images and evaluated on raw k‐space acquisitions.

**Study Type:**

Prospective feasibility study.

**Population:**

Briefly, 1289 male patients (aged 44–87, median age 68) for model training, 8 male patients (aged 59–78, median age 65) for prospective feasibility testing.

**Field Strength/Sequence:**

2D Cartesian balanced steady‐state free precession, 3 T.

**Assessment:**

Segmentation and reconstruction models were trained on 8464 MRGB confirmation scans containing a biopsy needle guide instrument and evaluated on 10 prospectively acquired dynamic k‐space samples. Needle guide tracking accuracy was assessed using instrument tip prediction (ITP) error, computed per frame as the Euclidean distance from reference positions defined via pre‐ and post‐movement scans. Feasibility was measured by the proportion of frames with < 5 mm error. Additional experiments tested model robustness under increasing undersampling rates.

**Statistical Tests:**

In a segmentation validation experiment, a one‐sample *t*‐test tested if the mean ITP error was below 5 mm. Statistical significance was defined as *p* < 0.05. In the tracking experiments, the mean, standard deviation, and Wilson 95% CI of the ITP success rate were computed per sample, across undersampling levels.

**Results:**

ITP was first evaluated independently on 201 fully sampled scans, yielding an ITP error of 1.55 ± 1.01 mm (95% CI: 1.41–1.69). Tracking performance was assessed across increasing undersampling factors, achieving high ITP success rates from 97.5% ± 5.8% (68.8%–99.9%) at 8× up to 92.5% ± 10.3% (62.5%–98.9%) at 16× undersampling. Performance declined at 18×, dropping to 74.6% ± 33.6% (43.8%–91.7%).

**Data Conclusion:**

Results confirm stable needle guide tip prediction accuracy and support the robustness of the reconstruction model for tracking at high undersampling.

**Evidence Level:**

2.

**Technical Efficacy:**

Stage 2.

## Plain Language Summary

Prostate biopsies guided by MRI are accurate but slow. This paper discusses a method to speed it up using artificial intelligence (AI). The method creates medical images from less data, allowing for the tracking of the biopsy needle guide tip in near real‐time. The AI was trained using 8464 scans of 1289 men and was then tested in 8 patients. Success was defined as the tip being within 5 mm of its actual position. The method stayed accurate with as little as one‐sixteenth of the usual data. Such speed‐ups could shorten procedures and free up scanner time.

## 1 Introduction

Prostate cancer is the second most commonly diagnosed cancer in men [1]. Following an abnormal digital rectal exam or elevated prostate‐specific antigen levels, the current standard of care is to perform a multiparametric MRI of the prostate, often followed by an MRI‐targeted biopsy if suspicious lesions are identified [2, 3]. Three MRI‐targeted biopsy techniques are common: ultrasound‐guided cognitive biopsy, where the clinician mentally maps MRI‐identified lesions onto ultrasound; software‐assisted MRI‐ultrasound fusion, which aligns and overlays images for targeting; and in‐bore MR‐guided biopsy, performed in the scanner with images after each needle guide position to confirm and refine targeting. Evidence remains inconclusive to determine whether any technique has an advantage in cancer detection [4, 5, 6]. However, in‐bore MR‐guided biopsy (MRGB) allows needle position confirmation and precise targeting of small or anatomically difficult lesions, making it well suited for complex cases. Still, its broader adoption is limited by longer procedure times and higher resource demands, largely due to the need for repeated imaging for needle guide verification and manual adjustments [7].

Addressing these limitations in MRGB requires technical innovation. Faster MR acquisition could reduce the delays between targeting and confirmation, thereby streamlining the workflow. Accurately reconstructing undersampled acquisitions shortens scan time, which may enable real‐time instrument tracking and reduce instrument guidance time. Furthermore, cancer detection rates in prostate biopsy appear mostly influenced by operator experience [8]. By simplifying targeting and minimizing manual steps, these advances may make the procedure easier to perform and, in turn, improve cancer detection.

Developments in iterative reconstruction techniques have been used to reconstruct real‐time MR acquisitions, and iterative methods have been applied to cardiac catheter instrument guidance [9, 10]. More recently, deep learning‐based image reconstruction methods have been applied to real‐time cardiac MRI and have since been extended to other procedures, including MR‐guided neurosurgery and MR‐guided radiotherapy [11, 12, 13]. However, these studies focus solely on image reconstruction and often rely on simulated k‐space data (e.g., introducing artificial phase) or simulated instruments (e.g., using phantoms), which limit their ability to accurately reflect real‐world clinical conditions.

To address these limitations and better reflect clinical reality, this work was centered on instrument tip localization during MRGB under accelerated acquisition. It has been reported that MR‐guided robotic biopsy achieves a mean error of 2.5 mm with a standard deviation of 1.6 mm, and that errors above 5 mm risk missing targets [14]. The goal of this study is to extend deep learning image reconstruction to MRGB, assess whether it preserves clinically acceptable instrument tip localization at accelerated acquisition, and characterize the effects of higher accelerations on real‐time guidance.

## 2 Materials and Methods

### 2.1 Training Data

The use of retrospective data for this study was approved by the institutional review board at our center (identifier: CMO 2016‐3045, Project 20,011). Informed consent was deemed exempt due to the retrospective scientific use of de‐identified patient data, and all patient data from January 2014 to December 2022 were consecutively included. All data were acquired on a 3 T scanner (MAGNETOM Skyra, Siemens Healthineers, Erlangen, Germany) with standard body and spine phased array coils. The imaging protocol, a balanced steady‐state free precession sequence (bSSFP), and its parameters used during these acquisitions are summarized in Table 1.

**TABLE 1:** MRI dataset imaging parameters.

	Pre‐ and post‐movement scans	Accelerated dynamic scans
Sequence type	2D bSSFP	2D bSSFP
Orientation	Transverse oblique	Transverse oblique
TR/TE	4.56 ms/2.28 ms	4.68 ms/2.34 ms
Flip angle	70°	70°
Field of View (mm)	280 × 280	280 × 280
Matrix (frequency × phase)		
Encoded	256 × 320	256 × 320
Reconstructed	256 × 256	256 × 256
Phase oversampling	25%	25%
Number of acquired phase‐encoding lines	320	68
Effective undersampling rate	1×	4.7×
In‐plane resolution (mm)	1.094 × 1.094	1.094 × 1.094
Slice thickness (mm)	3	3
Number of slices	5	1
Number of dynamic frames	1	60
Scan time	7.30 s	19.09 s (0.32 s per frame)

Abbreviations: bSSFP: balanced steady state free precession, TE: echo time, TR: repetition time.

The 2‐dimensional (2D) bSSFP DICOM dataset comprised 8464 scans from 1289 male patients (aged 44–87, median age 68) suspected of having prostate cancer. These patients underwent transrectal MRGB with a transrectal needle guide operated either via a manual aiming device (Invivo, Schwerin, Germany) or a remotely controlled manipulator system (Soteria Medical, Arnhem, Netherlands) [15, 16]. The images captured the pelvis, prostate, and the transrectal needle guide, with the image plane aligned along the needle guide axis to display the needle guide feature.

### 2.2 Deep Learning‐Based Temporal Image Reconstruction Model

The Convolution Recurrent Neural Network (CRNN‐MRI) model was adopted for iterative reconstruction of dynamic MR images due to its efficiency and simplicity in handling spatiotemporal data [17]. The model introduces recurrence across iterations and time, enabling shared learning of temporal dynamics. The model architecture comprises three convolutional recurrent units (CRNN) that evolve over iterations and one bi‐directional CRNN that evolves across time and iterations.

The publicly available implementation (github.com/js3611/Deep‐MRI‐Reconstruction) was adapted by modifying the original pixel‐wise mean squared error loss to optimize the structural similarity index measure instead, which better preserves both anatomical structures and the appearance of the needle guide. All other hyperparameters were kept consistent with the “Proposed‐B” configuration described in [17], using 10 iterations with five components per iteration: one bi‐directional CRNN unit, three CRNN units, and one CNN unit, each with a kernel size of 3 and 128 filters.

To simulate undersampled k‐space data, fully sampled post‐processed DICOM images were first transformed into the frequency domain using a two‐dimensional Fourier transform. Synthetic undersampling was then performed by applying a binary mask that retained only the central lines of k‐space, effectively zeroing all peripheral lines. This approach reflects the distribution of retained lines in the evaluation dataset. Image intensities were scaled to the 0–1 range, and data augmentation included random horizontal and vertical flipping, rotations by multiples of 90 degrees, and additional Rician noise to better simulate MRI conditions.

### 2.3 Self‐Configuring Deep Learning‐Based Image Segmentation Model

The needle guide was segmented from reconstructed images by training a baseline nnU‐Net model [18]. This U‐Net‐based framework automatically configures its architecture and training parameters to match the characteristics of the dataset. The nnU‐Net's default trainer and planner with the 2D configuration was used.

1009 scans from 203 randomly selected patients in the DICOM dataset were extracted. Patients were randomly selected, and all associated scans were included until the scan count exceeded 1000. Annotations were performed on the central slice of each scan by marking a line from the base to the tip of the visible needle guide. This work was carried out by C.R.N. under the supervision of J.J.F. and K.O. (20 and 12 years of experience in prostate biopsies, respectively). The annotated line defined the axis of a hypothetical cylindrical region, and all voxels intersecting this region were included in the segmentation mask.

### 2.4 Principal Component Analysis

To estimate the location of the needle guide tip, principal component analysis (PCA) was applied to the segmentation mask, following the approach described in [19]. First, the centroid of the segmented region was calculated. Then, the spatial coordinates of the segmentation are used to compute a covariance matrix, from which the principal eigenvector is extracted to represent the needle guide's main axis. The tip position was defined as the endpoint of this axis that lies closest to the center of the image.

### 2.5 Experimental Setup

The performance of the segmentation model was first validated independently. The annotated subset of scans used for segmentation model development was randomly split into training and validation sets using an 80/20 ratio.

To evaluate the feasibility of the real‐time image reconstruction model for accurate needle guide tip prediction, a series of tracking experiments was conducted using a prospective dataset of raw k‐space acquisitions. Between June 2023 and January 2025, 22 male patients (aged 57–78, median age 70) suspected of having prostate cancer and scheduled for MRGB procedures were recruited on occasion. The experimental component of this study was approved by METC Oost‐Nederland (2023‐16496), and informed consent was obtained from each participant prior to inclusion.

**FIGURE 1:**
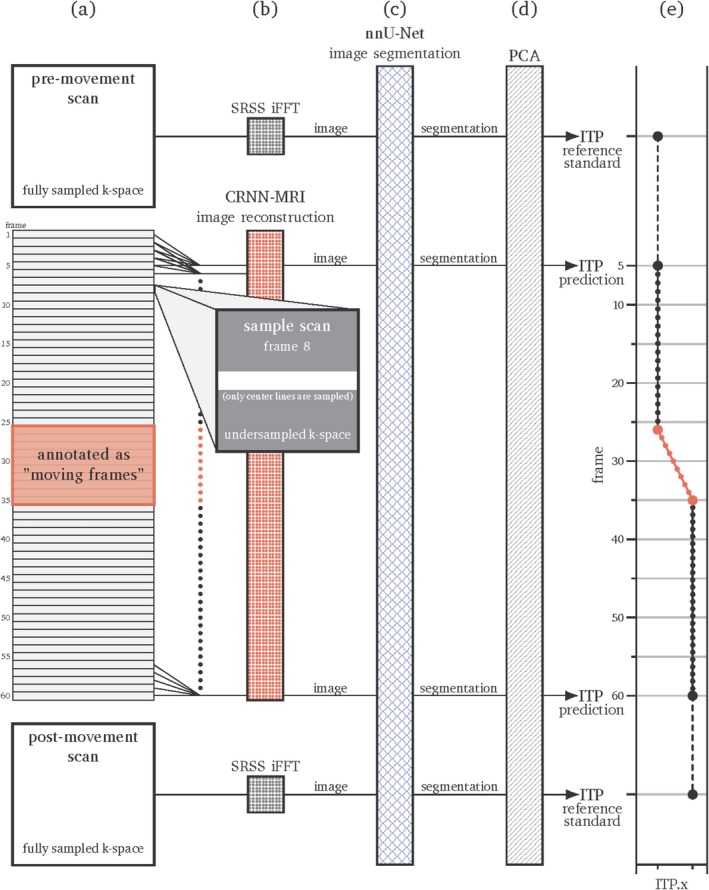
Setup for the tracking experiment. (a) Each sample included pre‐, dynamic, and post‐movement scans; the figure shows an example range of frames annotated where instrument motion was observed. Frame 8 is highlighted to illustrate the undersampling pattern: Gray denotes unsampled k‐space and white denotes sampled k‐space. (b) Pre‐ and post‐movement scans were reconstructed using root sum of squares coil combination followed by 2D inverse Fourier transform (SRSS iFFT); dynamic frames were undersampled and reconstructed using an image reconstruction model (CRNN‐MRI) operating on a sliding window of five frames. (c, d) Segmentations were output for all frames, and PCA was applied to estimate the instrument tip position (ITP) per frame. (e) A reference trajectory was computed by interpolating between the reference ITP from the pre‐ and post‐movement segmentations over the annotated frames.

Each sample included a pre‐movement scan, a sequence of 60 frames acquired during needle guide movement, and a post‐movement scan (Figure 1a). The pre‐ and post‐movement scans were fully sampled 2D multi‐slice Cartesian bSSFP scans (Table 1), reconstructed using a two‐dimensional inverse Fast Fourier Transform followed by root sum of squares combination of the individual coil images. The dynamic Cartesian bSSFP scans were acquired with identical settings for field of view and matrix, but with a generalized auto‐calibrating partial parallel acquisition (GRAPPA) factor of 8, using 32 autocalibration signal (ACS) lines per frame. Only the 32 ACS lines were retained and zero‐padded to a matrix of 256 × 256. These data were then passed through the image reconstruction model, which operated on a sliding window of five frames to generate each reconstructed image (Figure 1b). The 32 ACS lines represent an undersampling of 8×. Higher accelerations can be obtained by zeroing additional outer ACS lines. For example, to achieve 16× undersampling, the 8 top and bottom ACS lines were set to zero.

All images were segmented using the trained segmentation model. For these experiments, it was assumed that the pre‐ and post‐movement segmentations were sufficiently accurate to define as reference start and end positions of the needle guide tip (Figure 1c). PCA was applied to the resulting segmentations to estimate the tip location for each frame (Figure 1d).

**FIGURE 2:**
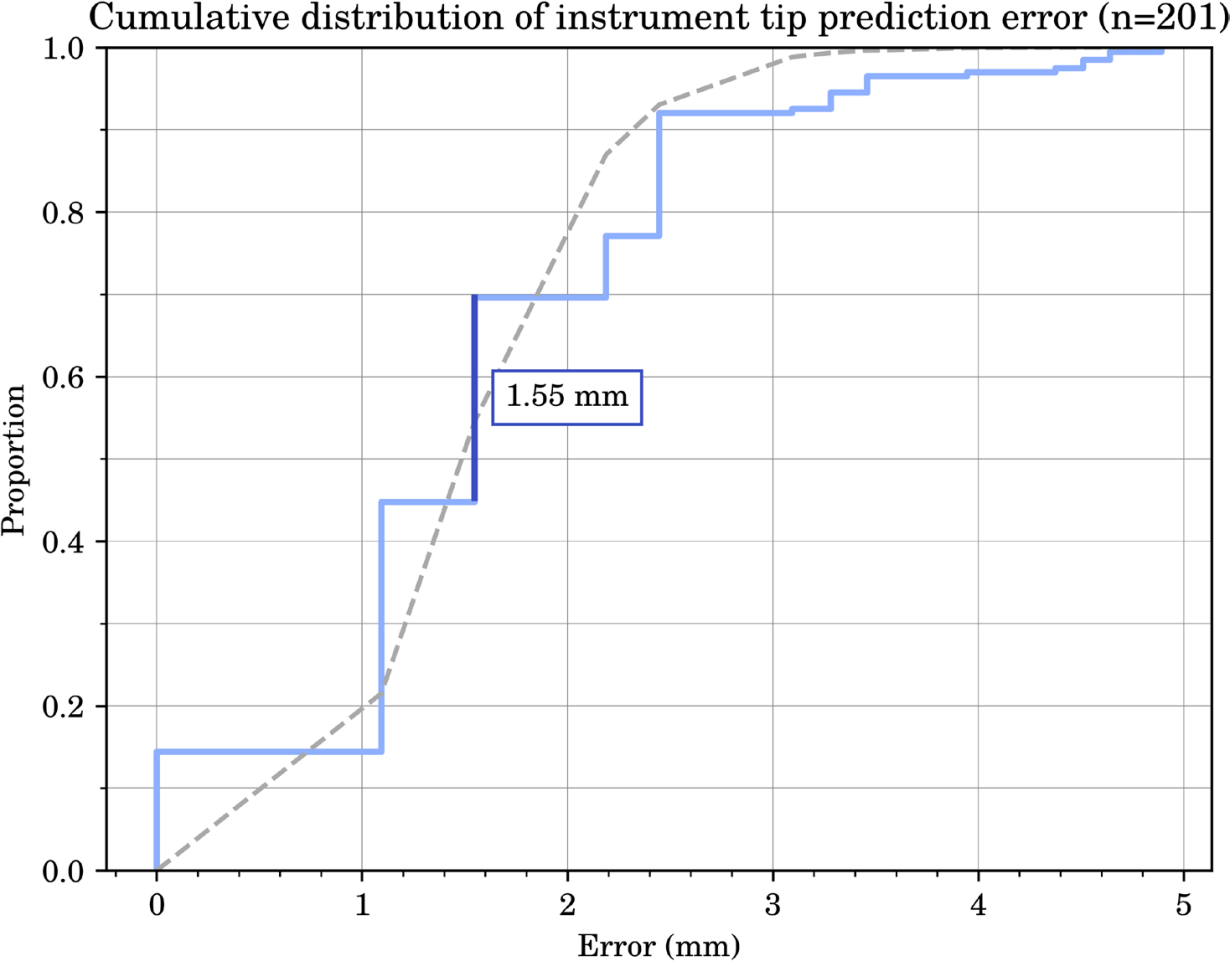
Empirical cumulative distribution plot of instrument tip prediction error from the segmentation algorithm, evaluated on 201 samples. The orange segment indicates the mean error in mm. A dashed line shows a fitted gamma distribution (shape = 7.64, scale = 0.20) for reference.

A reference trajectory was generated by linearly interpolating between the tip positions from the pre‐ and post‐movement segmentations across the annotated frames (Figure 1e). The time interval during which the needle guide was in motion (e.g., frames 26–36 in Figure 2a) was manually annotated. Additional experiments were conducted using synthetically increased undersampling by further removing ACS lines. Frames with missing ACS lines or otherwise unreadable were excluded, but the samples were retained if both pre‐ and post‐movement scans remained intact after exclusion.

### 2.6 Evaluation Metrics

The primary evaluation metric for tracking performance was the needle guide instrument tip prediction (ITP) error, defined as the Euclidean distance in millimeters between the predicted tip position and the reference tip position for each frame. Guided by prior MR‐guided biopsy accuracy data [14], a 5 mm threshold was applied: success if < 5 mm and failure if ≥ 5 mm. For the segmentation validation experiment, where samples are independent, the ITP error is reported. For the needle guide tracking experiments, each sample from the k‐space dataset consists of 60 sequential and statistically dependent frames; the mean ITP success rate per sample, defined as the proportion of frames with an error below 5 mm, is reported. No special consideration to the annotated moving frames was given.

### 2.7 Statistical Analysis

For segmentation model validation, a one‐sample Student's t‐test was performed to evaluate whether the mean ITP error was significantly below 5 mm. Although ITP error is a non‐negative measure, the large sample size justified the assumption of approximate normality required for the t‐test. A standard t‐based confidence interval (CI) was computed for the sample mean, and a *p* value < 0.05 was considered significant. A fit of $\text{Gamma}\left(\alpha ,\theta \right)$ was obtained by moment matching to sample skewness, γ, and mean $\bar{x}$ ($\alpha =4/\gamma^{2}$, $\theta =\bar{x}/\alpha$). For the tracking experiments, the mean, standard deviation, and 95% Wilson score CI of the per‐sample ITP success rate were computed at increasing undersampling levels. Analysis was conducted using Python (python‐v3.10, scipy‐v1.15).

## 3 Results

Segmentation performance was first evaluated independently to ensure it would not confound downstream tracking evaluation. In total, 1002 out of 1009 scans were successfully annotated; the remaining scans were excluded due to the absence of a visible needle guide. On the validation set of 201 annotated, fully sampled scans, the model achieved a mean instrument tip prediction error of 1.55 ± 1.01 mm (95% CI: 1.41–1.69). The 95% CI lies between the next two possible error values, given the voxel resolution of the MRI scan, suggesting the population mean is likely equal to the observed sample mean. The error distribution was positively skewed with kurtosis = 4.11, consistent with a fitted gamma distribution (Figure 2).

**FIGURE 3:**
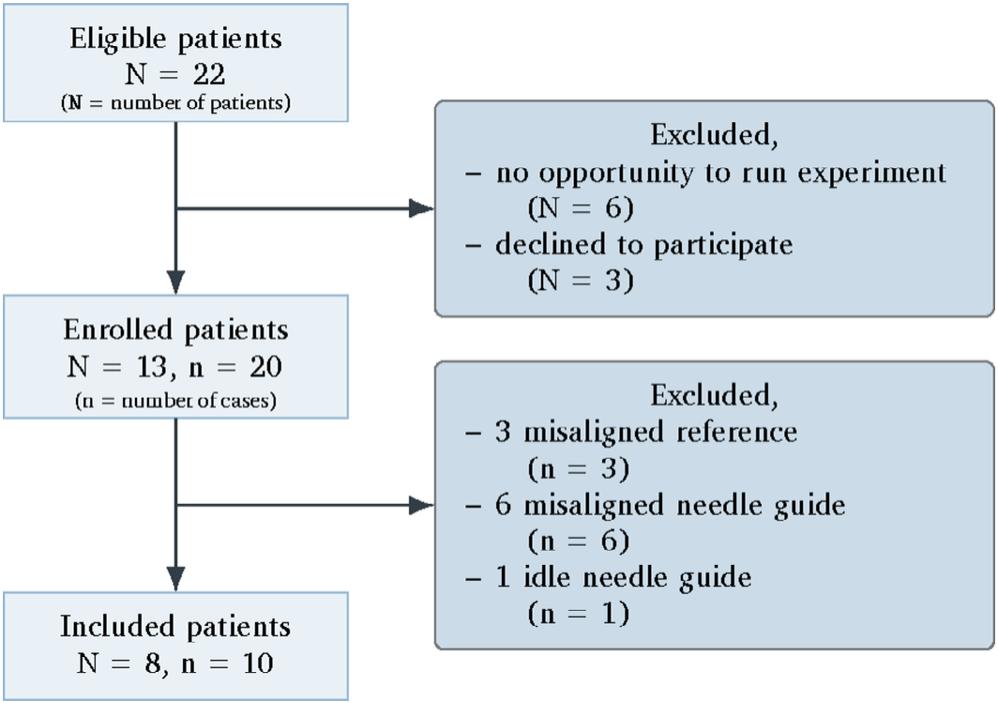
STARD flow diagram, demonstrating the participant selection process for this study. Eight participants were ultimately included, providing 10 samples.

A total of 20 raw 2D dynamic accelerated Cartesian bSSFP k‐space samples were collected during these procedures. Half of the samples were excluded due to misalignments, data corruption, or unexpected artifacts. Sample assessment was performed by C.R.N. under the supervision of J.J.F. (20 years of experience in prostate biopsies). A study flow diagram for this prospective dataset is provided in Figure 3.

The tracking performance under varying undersampling conditions is summarized in Table 2. At 8× and 10×, CRNN‐MRI reconstructions achieved high ITP success rates of 97.5% ± 5.8% (68.8%–99.9%) and 98.4% ± 3.6% (70.0%–99.9%), respectively, closely matching the zero‐filled results. As undersampling increased, CRNN‐MRI maintained viable tracking performance up to 16×, whereas zero‐filled reconstructions fell below viability from 12× onwards. At 12×, CRNN‐MRI achieved 97.9% ± 4.6% (69.3%–99.9%), compared to 60.4% ± 39.2% (31.6%–83.4%) with zero‐filled data. At 14×, the CRNN‐MRI success rate dipped to 83.0% ± 24.7% (52.1%–95.7%) before recovering to 92.5% ± 10.3% (62.5%–98.9%) at 16×, a notable deviation highlighted for completeness.

**TABLE 2:** Instrument tip prediction success rates across undersampling factors for zero‐filled and CRNN‐MRI reconstructions.

Undersampling		ITP success rate, % (mean ± standard deviation, (95% CI))	
*R*	No. of k‐lines	Zero‐filled reconstructions	CRNN‐MRI reconstructions
8×	32	97.5% ± 5.5% (68.8%–99.9%)	97.5% ± 5.8% (68.8%–99.9%)
10×	25	94.8% ± 9.4% (65.3%–99.4%)	98.4% ± 3.6% (70.0%–99.9%)
12×	21	60.4% ± 39.2% (31.6%–83.4%)	97.9% ± 4.6% (69.3%–99.9%)
14×	18	42.1% ± 36.3% (18.2%–70.4%)	83.0% ± 24.7% (52.1%–95.7%)
16×	16	34.6% ± 33.8% (13.5%–64.3%)	92.5% ± 10.3% (62.5%–98.9%)
18×	14	9.6% ± 12.3% (1.7%–40.0%)	74.6% ± 33.6% (43.8%–91.7%)
20×	12	9.6% ± 17.8% (1.7%–40.0%)	60.9% ± 24.0% (32.0%–83.7%)
25×	10	12.9% ± 20.4% (2.8%–43.6%)	8.6% ± 9.2% (1.4%–38.8%)

Abbreviations: CI: confidence interval; CRNN: convolutional recurrent neural network; ITP: instrument tip prediction; R: acceleration factor.

**FIGURE 4:**
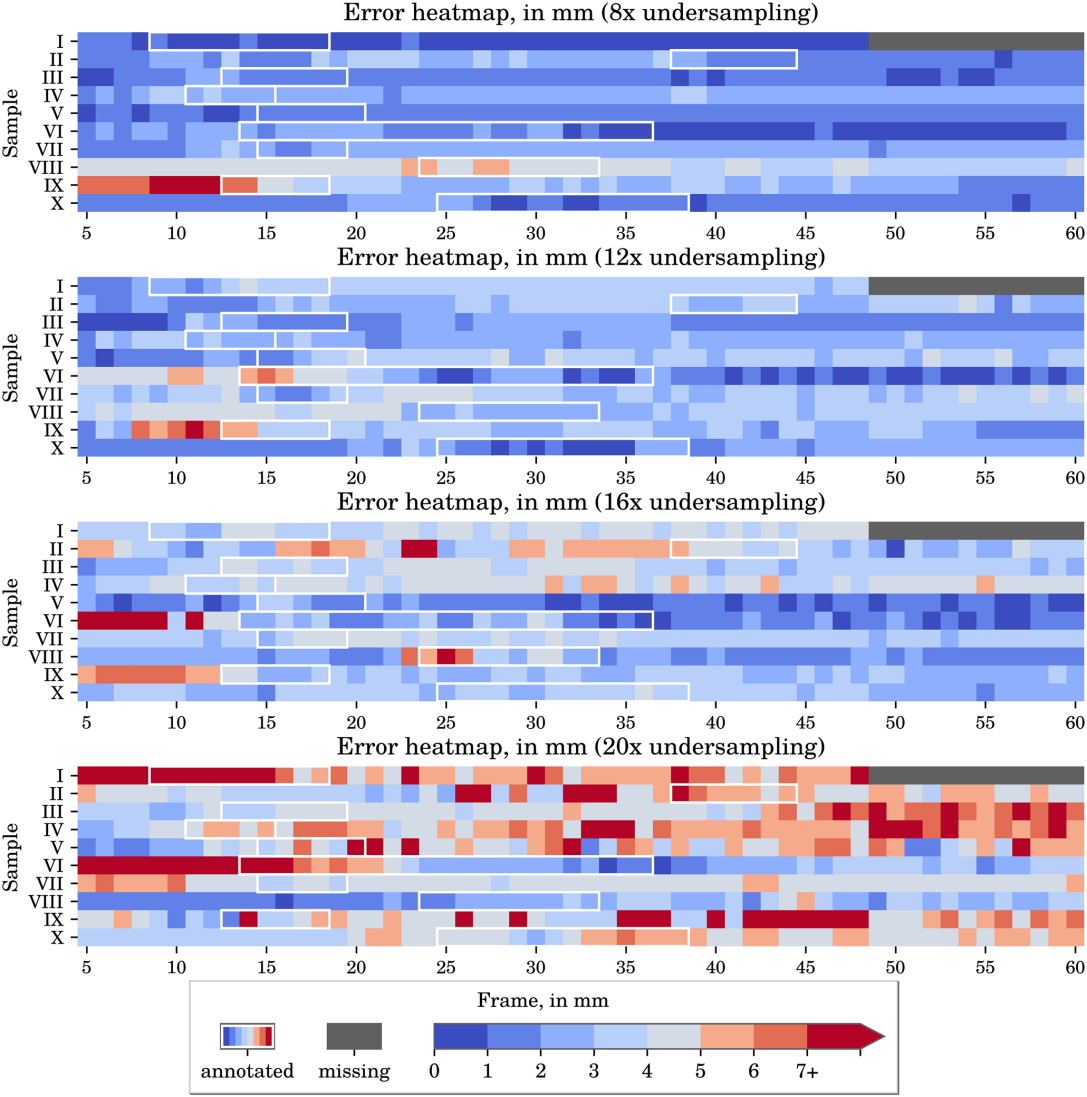
Instrument tip prediction error heatmap across all frames for selected undersampling levels, using CRNN‐MRI reconstructions. White outlines indicate annotated frames during which the instrument was in motion. Prediction errors greater than 5 mm are considered failures and are shown in shades of red, while prediction errors equal or less than 5 mm are considered successes and are shown in shades of blue.

Figure 4 shows frame‐by‐frame ITP error heatmaps across all samples at selected undersampling factors using CRNN‐MRI reconstructions. Each row corresponds to a sample, and each column corresponds to a frame, with the annotated movement frames outlined in white. As undersampling increases, cells shaded in red become more prominent, indicating a steady increase in the number of inaccurate predictions. In sample I, the final 12 frames were excluded due to unreadable data, but the pre‐ and post‐movement scans remained intact, so the sample was retained.

**FIGURE 5:**
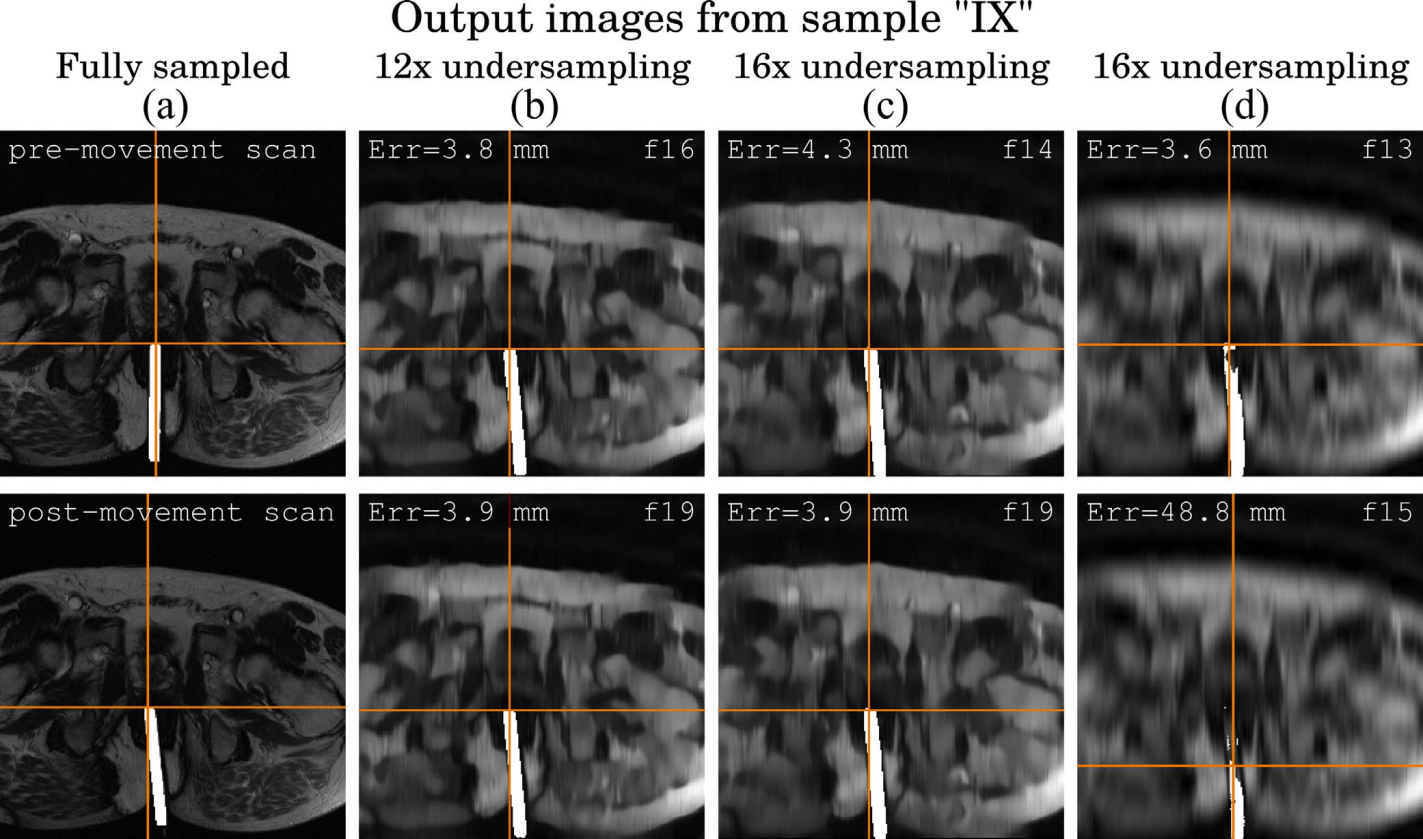
Example image reconstructions and segmentation masks for sample IX. (a) Fully sampled pre‐ and post‐movement scans. The remaining columns show predicted instrument tips, each labeled with the corresponding prediction error and frame number. (b, c) at 12× and 16× undersampling respectively, using CRNN‐MRI image reconstructions, (d) at 16× undersampling, using zero‐filled image reconstructions.

Figure 5 presents sample reconstructions and segmentation results for sample IX. The first column shows the fully sampled pre‐ and post‐movement scans used to define the reference trajectory. Subsequent columns display frames with predicted instrument tip locations, annotated with prediction error and frame number.

## 4 Discussion

This study demonstrates that a spatiotemporal deep learning framework may enable accurate and consistent tracking of a needle guide during MRGB, maintaining ITP errors below the clinically relevant 5 mm threshold [14]. After isolating reconstruction performance from segmentation accuracy, the results indicate that the CRNN‐MRI image reconstruction network, paired with a nnU‐Net segmentation network, supports robust tracking performance at acceleration factors of up to 16×.

While zero‐filled reconstruction is not a conventional baseline, it is a useful tool to highlight CRNN‐MRI's ability to preserve tracking accuracy under degraded input conditions. Zero‐filled reconstructions quickly became unreliable; the image reconstruction model maintained viable performance at higher acceleration levels. These observations are consistent with a prior study on the benefits of recurrent neural networks for dynamic reconstruction [20]. These results build on this by demonstrating their relevance in the context of real‐time, task‐driven instrument tracking during MR‐guided prostate biopsy.

These results can be positioned alongside other recent efforts toward deep learning–based real‐time navigation with lesion‐level accuracy for interventional radiology. In ultrasound, a spatiotemporal network achieved real‐time needle tracking and segmentation during breast biopsy, addressing a different anatomy [21]. Deep learning‐based real‐time tracking of probe orientation during freehand transperineal biopsy enabled cognitive navigation without external sensors [22]. These findings extend this trend to MRGB by demonstrating that a temporally aware image reconstruction model can support reliable device tracking at high acceleration, addressing an important bottleneck without external trackers or sensors.

Zero‐filled reconstruction was not intended as a clinically viable baseline but served as a reference to contextualize the performance of CRNN‐MRI. A noted dip in performance at 14× undersampling, which did not persist at 16×, may reflect sensitivity to specific cases or temporal patterns. In particular, individual samples where the ITP error was unexpectedly higher than in the same sample at higher levels of acceleration were observed. Although tracking performance generally declined with increased undersampling, these outliers show that this trend is not strictly consistent and may reflect model sensitivity to specific spatial or temporal patterns. Wilson score CIs consistently showed low lower bounds across the dataset, even when mean success rates were high. This behavior is a known property of the Wilson score interval in settings with high success rates and small sample sizes, where the lower bound is adjusted downward to account for binomial uncertainty rather than indicating increased uncertainty in the model's performance.

## 5 Limitations

The primary limitations of this study relate to the dataset composition and imaging protocol. Although the dataset was prospectively acquired and annotated, the number of unique procedures and subjects was limited, which may affect generalizability. The evaluation was restricted to 2D Cartesian bSSFP sequences. Other sequences, such as radial or stack‐of‐stars acquisitions, might be more robust to motion and undersampling artifacts and could offer advantages for instrument tracking [23, 24]. For example, a deep learning‐based model for MR‐guided in‐bore percutaneous needle interventions enabled automatic slice alignment in real time using undersampled radial k‐space spokes [25]. Furthermore, only a single reconstruction model architecture was evaluated; performance may vary with alternative models or different training strategies. Finally, the study was conducted at a single center using a single vendor system at a fixed field strength, which limited its generalizability.

Future research should investigate whether similar performance can be achieved prospectively in live scanner environments, across various anatomical targets, interventional instruments, and more suitable MR acquisition methods. Broader validation across centers and operators will also be necessary to assess the generalizability and robustness of the findings. If the framework is further tested and validated, integrating this into a clinical workflow through scanner‐side deployment will be a key step toward translating these technical advances into tangible clinical benefits.

## 6 Conclusion

The spatiotemporal deep‐learning reconstruction demonstrated reliable needle‐guide‐tip tracking at acceleration factors up to 16× k‐space undersampling, with no loss of accuracy. This supports real‐time image updates during MRGB prostate interventions and reduces acquisition time, which in turn is expected to shorten overall procedure duration and lower associated costs. These gains strengthen the clinical feasibility of incorporating MRGB into routine practice, and future in vivo studies will quantify the workflow and economic impact.
